# Drug-induced plasma discolouration mimicking icterus risks inappropriate withholding of critical lactate

**DOI:** 10.1016/j.plabm.2026.e00543

**Published:** 2026-06-03

**Authors:** Briedgeen Kerr, Aine Murphy, Conal Houstoun, Janet Tan, Vitaliy Mykytiv, Seán J. Costelloe

**Affiliations:** a Department of Clinical Biochemistry, Cork University Hospital, Cork, Ireland; b Department of Haematology, Cork University Hospital, Cork, Ireland; c Department of Medicine, University College Cork, Cork, Ireland

**Keywords:** Lactate, Icterus, Interference, Eltrombopag, Photometry, Fluoride–oxalate, Sepsis

## Abstract

A lactate concentration ≥4 mmol/L is associated with organ dysfunction and poor outcomes in sepsis, making accurate measurement and timely communication of critical results vital. Photometric lactate assays are common but prone to spectral interference from icterus, and objective assessment of haemolysis/icterus/lipaemia indices is essential to prevent release of compromised results. The authors report a patient with severely elevated lactate (10.2 mmol/L) in fluoride–oxalate plasma. The specimen was run on a colourimetric lactate oxidase method (Beckman Coulter AU5800 analyser at Cork University Hospital (CUH)). Since icteric indices were not routinely determined for fluoride–oxalate specimens, the sample was assessed by eye to be grossly icteric, and the initial decision was to withhold the lactate result per manufacturer guidelines. On review by the Consultant Clinical Biochemist, an automated icteric index was requested, which yielded a 1+ flag, corresponding with a total bilirubin in the range 42.7–85.5 μmol/L, and inconsistent with gross icterus. Review of the patient history indicated aplastic anaemia. Drug–related discolouration of the sample was suspected, and the haematology team confirmed eltrombopag therapy. The lactate result and subsequent reports were released with advisory comments, noting that the degree of any eltrombopag-related analytical interference was uncertain. This case demonstrates how a combination of drug–induced plasma discolouration, reliance on visual assessment of icterus, and inconsistent application of HIL indices across specimen types jeopardises the release of critical lactate results. Standardised automated index testing, clear protocols for interference evaluation, and timely multidisciplinary discussion of medication history are important safeguards in clinical laboratory practice.

## Highlights

• Visual assessment of icterus in plasma is unreliable.
• Drug-induced plasma discolouration further compromises visual assessment of icterus.
• Icteric indices support the safe release of critical results, including lactate.
• Eltrombopag discolouration can confound lactate interference assessment.

## 1 Introduction

Preanalytical factors represent an increasingly well–characterised source of error within medical laboratory practice [[1], [2], [3], [4], [5], [6], [7]]. Laboratories have, over many years, developed robust standard operating procedures to identify and mitigate against errors in the preanalytical phase. Interference arising from endogenous constituents, such as free haemoglobin, lipids, and bilirubin, or exogenous agents, such as drugs or poisons, is a significant cause of preanalytical variability that can erroneously affect the quantitation of analytes in biological specimens.

Automated HIL indices reduce reliance on subjective visual assessment, which is non-standardised and prone to misclassification when non-bilirubin chromogens cause plasma discolouration.

The Irish National Guidelines on Sepsis Management for Adults [8] state a lactate cutoff of >4 mmol/L as evidence of organ dysfunction, with a high associated mortality rate. Both National and International guidelines for the management of sepsis and septic shock recommend using lactate measurements to guide resuscitation in patients with elevated lactate (>2 mmol/L) [8,9]. Lactate is, therefore, routinely measured in critically ill patients, as it has significant diagnostic and prognostic value. The importance, therefore, of generating analytically accurate results, given their impact on patient management and care, cannot be overstated.

The case described illustrates a specific patient-safety risk arising when drug-induced plasma discolouration is mistaken for gross icterus within a workflow that relied on visual assessment of fluoride–oxalate specimens rather than automated HIL indices. Rather than highlighting only the well-recognised limitations of visual HIL grading, this report shows how a specimen that appeared grossly icteric by eye could have led to inappropriate withholding of a critical lactate result.

## 2 Case and laboratory investigation

### 2.1 Local workflow and interference assessment

Blood for lactate analysis in the laboratory at CUH is collected into fluoride–oxalate preservative to inhibit glycolysis and prevent in vitro lactate production from pyruvate. Use of this preservative helps minimise the generation of erroneously high lactate results, particularly when specimens are delayed in separation, as is common in primary care specimens. Lactate measurement on the Beckman Coulter AU5800 analyser uses an enzymatic photometric method in which lactate oxidase converts lactate to pyruvate and hydrogen peroxide (H2O2). Peroxidase then catalyses a reaction between H2O2, 4–aminoantipyrine, and a hydrogen donor to generate a quinonemine chromophore. Absorbance is measured at a primary wavelength of 540 nm with a secondary wavelength of 700 nm, and the resulting signal is directly proportional to the lactate concentration in the sample.

Icteric interference in this assay may arise through both spectral and chemical mechanisms [10]. Spectrally, bilirubin absorbs in the visible region and may contribute to background absorbance. Chemically, bilirubin can act as a reducing substance within the oxidase/peroxidase colour-forming system, decreasing dye formation and producing a negative bias. The manufacturer states that bilirubin interference is less than 10% or 0.25 mmol/L up to 8 mg/dL (137 μmol/L) bilirubin (icteric index 2+; Table 1) [11]. These claims were not independently verified locally in the present study. Published evaluation data on the AU5800 have suggested relatively limited negative bias at higher bilirubin concentrations, including reported bias of 7.4% in one study, although interpretation depends on the study design, analytical method, and performance criteria applied [12].

**Table 1:** **Interference limits and instrument flags for lactate and glucose on the AU5800.** Bilirubin (icterus index, ICTI), haemoglobin (haemolysis index, HI) and intralipid (lipemia index, LIPI) interference limits and associated flags are taken from the manufacturer's instructions for use.

Lactate interference by HIL on Beckman Coulter AU5800		
Interferent	Interference value	Instrument Flag
Bilirubin (icterus)	No significant interference up to 137 μmol/L	2+
Haemoglobin (haemolysis)	No significant interference up to 5 g/L	5+
Intralipid (lipaemia)	No significant interference up to 10 g/L	5+

Glucose interference by HIL on Beckman Coulter AU5800		
Interferent	Interference value	Instrument Flag
Bilirubin (icterus)	No significant interference up to 684 μmol/L	5+
Haemoglobin (haemolysis)	No significant interference up to 5 g/L	5+
Intralipid (lipaemia)	No significant interference up to 7 g/L	5+

An automated semi–quantitative photometric detection method for HIL is implemented on the Beckman AU series analysers used at CUH [13]. Absorbance is measured at multiple wavelengths (410 nm, 480 nm, 570 nm, 600 nm, 660 nm, 800 nm), and interference flags are generated for HIL based on the presumed presence and concentration of free haemoglobin, bilirubin, or lipid, as relevant. Different flags form an ordinal scale of increasing HIL severity for each interferent (normal (N), 1+, 2+, 3+, 4+, 5+, or abnormal (ABN)). These indices provide a semi-quantitative estimate of interferent type and severity and can be used to withhold or qualify results at risk of clinically significant HIL interference. Per the manufacturer's instructions, the integrity of all indices should be visually verified when ABN flags are observed on the Beckman AU system.

Historically, fluoride–oxalate samples at CUH showed a persistently high frequency of ABN HIL flags, particularly in specimens received from primary care in County Cork, where transport and processing delays were more common. On the Beckman AU system, an ABN flag still required visual inspection, as it could reflect either a spectral artefact without significant visible haemolysis or genuinely gross haemolysis. In practice, automated HIL assessment therefore did not remove the need for manual review in this specimen type. Because ABN flags occurred so frequently, visual inspection was required so often that the workflow effectively relied on manual adjudication. Although HIL indices continued to be generated, no electronic rule was in place at that time to incorporate these results into specimen handling or result release decisions for fluoride–oxalate samples.

In practice, this meant that for lactate testing, visual inspection was being used not to distinguish between minor gradations of icterus, but to decide whether a specimen appeared sufficiently icteric to raise concern for clinically significant interference and possible withholding of the result in line with the manufacturer's guidance. This was considered operationally acceptable because the relevant question for fluoride–oxalate analytes such as glucose and lactate was whether the specimen was grossly unsuitable for analysis, rather than the precise HIL grade. However, as the present case illustrates, such a workflow was vulnerable to misclassification when non-bilirubin chromogens, such as eltrombopag, caused marked plasma discolouration.

### 2.2 Index case and laboratory handling

In March 2022, a lactate concentration of 10.2 mmol/L (reference interval (RI): 0.5–2.2 mmol/L) was measured in a fluoride–oxalate sample on the Beckman Coulter AU5800 analyser in the Department of Clinical Biochemistry. The sample had been received from the Intensive Therapy Unit.

The icteric index was judged by eye, as per the laboratory protocol at that time, to be grossly icteric due to an observed red–brown discolouration of the plasma. The Scientist considered blocking the result as per the manufacturer's instructions. However, at the request of the Consultant Clinical Biochemist, an automated icteric index (as part of HIL indices) was performed and returned as ‘1+’, inconsistent with gross icterus, thereby excluding this as a valid reason for withholding the result. On review of the patient history, which showed a history of thrombocytopenia and aplastic anaemia, eltrombopag exposure was suspected as a cause of the plasma discolouration and confirmed with the haematology team. This was the only specimen in the episode initially judged visually to be icteric and considered for withholding on that basis. Following this event, laboratory staff were aware of the case and automated HIL indices were performed on subsequent specimens received from this patient.

The index and subsequent lactate specimens were collected into Greiner Bio-One fluoride–oxalate tubes. Other relevant specimens included Greiner Bio-One serum gel separator tubes for bilirubin, CRP, and routine biochemistry; Greiner Bio-One K3EDTA tubes for full blood count; Greiner Bio-One trisodium citrate tubes for coagulation studies; and lithium heparin whole-blood specimens for point-of-care analysis on the Siemens RapidPoint.

### 2.3 Clinical context and medication confirmation

The patient had severe aplastic anaemia treated with antithymocyte globulin and cyclosporine and was receiving eltrombopag 150 mg once daily. During the second week of rabbit ATG therapy, she was admitted to the intensive care unit (ICU) with severe neutropenic sepsis, at which time a lactate result of 10.2 mmol/L was obtained—the finding that prompted this short communication. Review of prior results showed persistently elevated bilirubin concentrations while on eltrombopag with otherwise unremarkable liver enzymes, and hepatology review favoured a drug–related explanation.

Eltrombopag is a thrombopoietin receptor agonist used to treat thrombocytopenia [[14], [15], [16], [17]]. As a highly coloured compound, it may cause both automated spectrophotometric interference in bilirubin assays and apparent visual icterus, owing to absorbance peaks at approximately 425 nm and 530 nm; these effects are method- and platform-dependent, including on the AU5800 analyser [[18], [19], [20]]. There is a paucity of published evidence on the interference of eltrombopag with lactate assays, which complicates the real–time interpretation of discoloured specimens.

### 2.4 Outcome

Based on further investigations and following a review of the case with the haematology team, along with an assessment of the patient's drug regimen, the lactate result and subsequent biochemistry reports were issued with advisory comments highlighting the presence of eltrombopag.

However, the magnitude and direction of any eltrombopag-related interference with this patient's plasma lactate result, total bilirubin measurement, or automated icteric index could not be determined retrospectively.

## 3 Discussion

The subjective assessment of icterus in the fluoride–oxalate specimen described in this report did not agree with the results of the automated measurement of the icteric index. This finding is consistent with previous studies comparing visual versus automated detection of HIL specimens, which found poor agreement between subjective and automated methods and poor intra–operator agreement in visual assessment [21]. It is estimated that approximately 50% of UK and Irish laboratories still use visual assessment alone or in combination with automated methods [7]. The reasons for this may be historical, due to a lack of automated HIL methods, a lack of governance, or limitations in workflow and automated HIL methodologies, as was the case in the episode described here.

Although the limitations of visual HIL assessment are well recognised, this case highlights an additional and distinct practical risk. In the local workflow described, visual inspection of fluoride–oxalate specimens was effectively being used as a pragmatic screen to identify gross interference sufficient to justify withholding lactate results, rather than to assign fine HIL gradations. Such an approach may appear operationally safe. However, in this case, drug-induced plasma discolouration created a false visual impression of gross icterus, thereby jeopardising the release of a critical lactate result.

Since eltrombopag-related discolouration can mimic icterus, it is easy to see how the practice of visual assessment of plasma might be misinterpreted as icterus, leading to withholding of an analytical measurement and posing a risk to patient safety. No photograph of the index specimen was retained; however, published images of eltrombopag-related serum/plasma discolouration are available elsewhere [20].

The broader issue is not confined to apparent icterus. Eltrombopag-related specimen discolouration may also confound assessment of haemolysis, whether by visual inspection or by automated serum-index analysis. In this respect, the present case reflects a wider preanalytical risk: strongly coloured drugs may distort interpretation of multiple HIL-related specimen quality checks, not only those directed at bilirubin-related interference [[22], [23], [24]].

Given the absorbance profile of eltrombopag, a positive bias in the icteric index would be expected if spectral interference were substantial; the low index observed therefore argues against marked icteric-index interference on this platform, despite obvious visual discolouration. However, in the absence of additional interference studies, this cannot be taken as proof that the icteric index or bilirubin measurement were unaffected by eltrombopag. This is in keeping with recent reports of visible eltrombopag-related discolouration with normal icteric index, and with the broader literature indicating that such interference is assay- and analyser-specific [[22], [23], [24]].

As the patient's bilirubin remained persistently elevated whilst on eltrombopag therapy, in the absence of other LFT abnormalities, the question remains whether eltrombopag interfered with the bilirubin assay itself. Bilirubin and eltrombopag have overlapping absorbance peaks at 425 nm, and eltrombopag also absorbs at 530 nm, within a similar measuring range as diazotised bilirubin [25]. Due to a lack of information regarding the patient's drug regimen and of laboratory processes to aid in identifying samples at risk of drug interference, this potential interference was not identified as a risk during processing numerous bilirubin requests for this patient in the Clinical Biochemistry laboratory.

Per the CLSI EP07 and EP37 guidelines [26,27], the effects of potential interferents on clinical chemistry results should be investigated, and their clinical significance evaluated. If the dose was taken at approximately 06:00, the elevated lactate was measured when eltrombopag levels were likely at or near peak, expected 2–4 h post–dose [28], although this cannot be confirmed from the available records. Eltrombopag has a plasma elimination half-life of approximately 21–32 h, is extensively metabolised, and is cleared predominantly via the faeces, with urinary excretion mainly as metabolites. In this context, drug-related sample discolouration may plausibly have been present in more than one specimen during treatment. However, the extent to which eltrombopag may have contributed to multiple lactate results cannot be determined retrospectively in this case. The red–brown discolouration of the sample, together with an icteric index of 1, was consistent with eltrombopag-related sample discolouration at the time of lactate measurement. Notwithstanding these findings, the clinical team considered the elevated lactate consistent with sepsis based on the patient's clinical presentation.

**Fig. 1:**
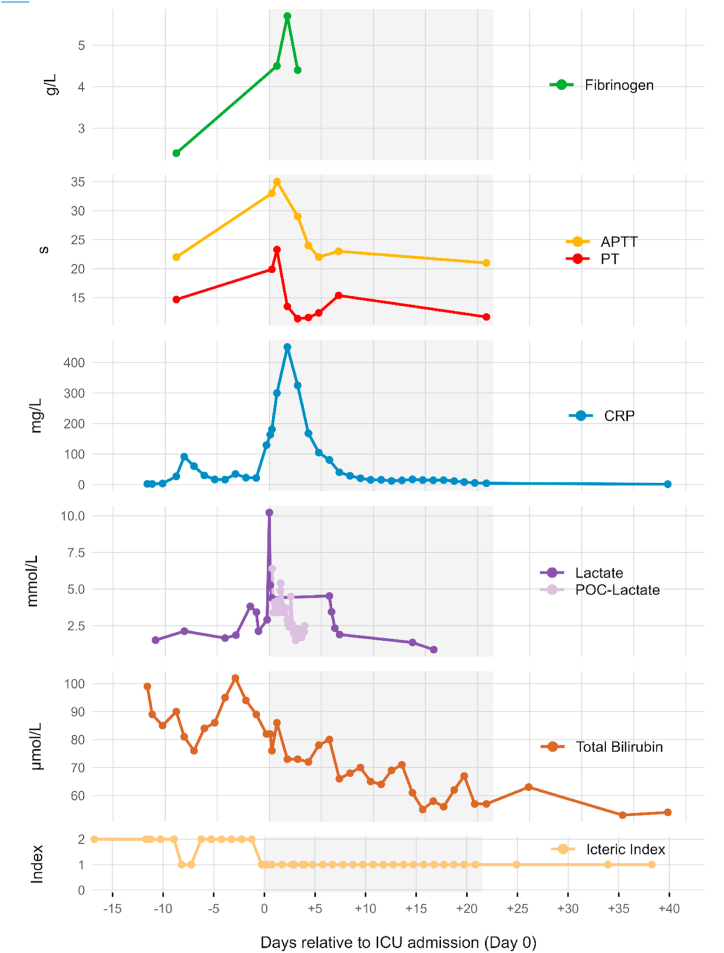
**Temporal trends in laboratory parameters relative to ICU admission (Day 0).** Fibrinogen, aPTT, PT, CRP, lactate (core laboratory and point-of-care), total bilirubin, and icteric index are shown across the pre-admission period, inpatient episode, and post-discharge follow-up. Eltrombopag treatment is indicated on the timeline.

An alternative lactate method was available (Siemens RapidPoint 500e), but no paired measurements were obtained at the time of peak lactate to assess method agreement. Later results were discordant (laboratory lactate 4.4 mmol/L at 21:15 from a 17:30 sample vs point–of–care lactate 6.4 mmol/L at 18:30), but interpretation was limited by non–synchronous sampling and preanalytical uncertainty (Fig. 1). This methodological difference is also relevant to the interpretation of interference. The Beckman AU5800 lactate assay is photometric and, in principle, susceptible to spectral interference from coloured compounds. In contrast, the Siemens RapidPoint lactate method uses lactate oxidase with amperometric detection and would not be expected to show the same form of colour-based spectral interference. However, no paired contemporaneous comparison was available at the time of peak lactate, so any method-specific effect of eltrombopag on the RapidPoint system could not be assessed directly in this case.

As discussed, there is clear evidence that eltrombopag can cause visible plasma discolouration and can interfere with some spectrophotometric chemistry assays, particularly bilirubin-related measurements and automated serum indices. However, published evidence specifically demonstrating interference with plasma lactate assays is lacking. Accordingly, the present case does not establish that eltrombopag interfered with the Beckman AU5800 lactate result. Rather, it shows that eltrombopag-related discolouration can mimic icterus and jeopardise release of a critical lactate result. Plasma lactate methods using photometric oxidase/peroxidase chromogenic detection may be vulnerable in principle to this type of effect, but the magnitude and direction of any true lactate interference remain uncertain.

No serial dilution studies or contemporaneous alternative-method comparisons were performed at the time of peak lactate. This limits interpretation of the extent to which eltrombopag may have contributed to interference in lactate, bilirubin, or automated serum indices in this case.

The point-of-care lactate measurements were performed on lithium heparin whole blood, and no formal HIL assessment was undertaken before analysis on the wards. More broadly, this reflects a limitation of many whole-blood POCT lactate workflows, in which formal preanalytical interference assessment was not routinely available at the time of this case. In practical terms, visual assessment of icterus in such specimens would require centrifugation and inspection of the separated plasma phase and is therefore not part of routine bedside testing. In the present case, where the discolouration was suspected to be drug-related rather than due to true marked icterus, such bedside visual assessment might instead have introduced the same risk of misclassification encountered in the core laboratory. Newer POCT systems have begun to incorporate integrated whole-blood haemolysis detection.

CRP increased concurrently with prolonged aPTT and PT and a rise in fibrinogen around the time of peak lactate, with subsequent improvement in these markers as lactate fell during ICU management (Fig. 1).

As discussed, there is certainly clear evidence that eltrombopag can interfere with spectrophotometric chemistry assays (e.g. bilirubin). However, published evidence is lacking regarding interference with plasma lactate measurement on the Beckman Coulter AU5800. Given this known impact on absorbance and the fact that the lactate assay is spectrophotometric, interference here remains a plausible concern and cannot currently be excluded from this case study.

Eltrombopag is increasingly used in immune thrombocytopenic purpura (ITP) and within haematology and is now incorporated into many guidelines [29,30]. An increase in the number of patients on eltrombopag may result in more specimens being received in the Clinical Biochemistry laboratory, with possible drug–related interferents and the potential for a specimen to be visually judged as icteric when it is not.

This case emphasises the importance of considering other factors when assessing discolouration of patient plasma. Consideration of the patient's history and clinical liaison allowed identification of the likely cause of serum discolouration. A different laboratorian may have adjudicated that the specimen was truly icteric and not released the critical lactate result.

The case demonstrates the need for precise clinical details, details of drug regimens, and reliable HIL indices to be run on all blood specimens, as well as for the assessment and documentation of how drugs interfere with biochemical measurements. If available, it is essential to ensure that analytes are measured by an alternative method for comparison purposes.

In addition, as automation advances, there is a need to identify discoloured specimens within an enclosed, fully automated tracking system. A laboratorian may never see the serum or plasma from these specimens, and they may not be identified during HIL analysis.

Fundamentally, laboratory staff need procedures for considering assay interferants that prioritise patient safety.

## 4 Conclusion

Drug–induced plasma discolouration can mimic icterus and undermine visual interference assessment, particularly where automated indices are not applied consistently across specimen types. For critical analytes such as lactate, reliance on visual inspection risks inappropriate withholding or delay of clinically important results. Standardised HIL indices testing, medication–aware interpretive protocols, and comparison by alternative methods where feasible are practical safeguards.

## Ethical approval and consent

Ethical approval was not sought as this report describes routine laboratory testing and service evaluation within standard clinical care and contains no patient–identifying information. Written informed consent for publication was obtained from the patient's parents and is documented in the patient record with supporting correspondence retained by the authors.

## Declaration of generative AI and AI-assisted technologies in the manuscript preparation process

During revision of this manuscript, the authors used ChatGPT (OpenAI) to assist with refining the manuscript structure, formatting, and language, and to optimise the R code used to generate Fig. 1. The authors also used Grammarly to support grammar and style review. After using these tools, the authors reviewed and edited all content as needed and take full responsibility for the content of the published article.

## Funding

This research did not receive any specific grant from funding agencies in the public, commercial, or not–for–profit sectors.

## CRediT authorship contribution statement

**Briedgeen Kerr:** Conceptualization, Data curation, Formal analysis, Investigation, Methodology, Visualization, Writing – original draft, Writing – review & editing. **Aine Murphy:** Data curation, Investigation, Resources, Writing – review & editing. **Conal Houstoun:** Investigation, Resources, Writing – review & editing. **Janet Tan:** Investigation, Resources, Writing – review & editing. **Vitaliy Mykytiv:** Investigation, Resources, Writing – review & editing. **Seán J. Costelloe:** Conceptualization, Formal analysis, Investigation, Methodology, Supervision, Visualization, Writing – original draft, Writing – review & editing.

## Declaration of competing interest

The authors declare that they have no known competing financial interests or personal relationships that could have appeared to influence the work reported in this paper.

## Data Availability

The data that has been used is confidential.
